# Sirtuin 3 in acute kidney injury

**DOI:** 10.18632/oncotarget.4794

**Published:** 2015-07-08

**Authors:** Luca Perico, Giuseppe Remuzzi, Ariela Benigni

**Affiliations:** IRCCS - Istituto di Ricerche Farmacologiche “Mario Negri”, Centro Anna Maria Astori, Science and Technology Park Kilometro Rosso, Bergamo, Italy

Twenty years of research and advances in pathophysiology have not yet translated into effective therapeutic tools for improving survival after an acute kidney injury (AKI) episode. AKI is a disease process characterised by rapidly decreasing renal excretory function, with accumulation of nitrogen metabolism and other waste products. AKI is a major public health concern with a rapidly increasing prevalence and unacceptably high mortality rate, claiming 1.7 million lives each year [1].

AKI is characterized by renal tubular damage, for which a plausible key mediator, amenable to pharmacological manipulation, has not been identified yet. Due to their high-energy demand, tubular cells are rich in mitochondria, and alterations in these organelles have been recognized as a hallmark in the initiation and progression of the disease [2]. Increased production of reactive oxygen species (ROS) and decreased antioxidant defences, make mitochondria particularly susceptible to injury [2]. These are highly mobile organelles that exist in dynamic networks whose function relies on complex molecular machinery, finely tuned and balanced between fission― accompanied by mitochondrial membrane depolarization and oxidative damage―and fusion, more commonly leading to energy production. Mitochondrial fission is catalyzed by Dynamin Related Protein 1 (Drp1), which is recruited from the cytosol to the outer mitochondrial membrane by Mitochondria fission factor (Mff) to form spirals to sever both membranes of the organelle. Conversely, mitochondria join through the process of fusion, which is mediated by the coordinated activities of Mitofusins (Mfn) and Optic atrophy 1 (Opa1) in the outer and inner mitochondrial membrane, respectively. Fission and fusion may also serve as an important quality control mechanism for preserving the functional integrity of the mitochondrial network. Fusion helps to ease physiological stress levels by mixing the contents of partially damaged mitochondria as a form of complementation, while fission is needed to remove mitochondria from the network when the organelles become damaged and membrane potential depolarization occurs across the inner membrane. Depolarized mitochondria are substrates for the translocation of several proteins, such as PTEN-induced Putative Kinase 1 (PINK1), which tags the organelle for elimination through the mitophagic machinery. Ample evidence indicates that, in experimental models of AKI, mitochondrial fission predominates and organelle impairment precedes renal dysfunction [3].

Mitochondrial vitality appears to be maintained by Sirtuin 3 (SIRT3), which belongs to an evolutionarily conserved family of seven NAD^+^-dependent deacetylases, three of which (SIRT3-5) are mainly localized in the mitochondrion [4]. In previous studies we demonstrated that elevated renal SIRT3 expression reduces ROS and ameliorates mitochondria dynamics that translate into the longevity phenotype in mice deficient for angiotensin II type 1 receptor [5]. Whether renal SIRT3 could be a master regulator of injury and repair in AKI represented a logical extension of these studies.

Cisplatin was used in mice to induce AKI that, by affecting proximal tubular cells, leads to increased generation of ROS and changes in mitochondrial dynamics [6]. These abnormalities were associated with an 80% reduction in the renal expression of SIRT3 mRNA and protein. In an attempt to pharmacologically manipulate the system, AICAR, an activator of the SIRT3 upstream signal AMPK, or the antioxidant agent acetyl-L-carnitine (ALCAR), were used. Both compounds improved renal function and ameliorated tubular injury in cisplatin-induced AKI mice. The renoprotective effect was accompanied by the upregulation of PGC-1α, a crucial mitochondrial biogenesis regulator able to promote SIRT3 gene expression, and NAMPT, the rate-limiting enzyme in the biosynthesis of NAD^+^, ultimately leading to restoration of SIRT3 expression/activity. At the ultrastructural level, AICAR treatment tipped mitochondrial dynamics towards fusion, as it preserved mitochondrial fragmentation induced by the chemotherapic and reduced expression of the mitochondrial fission mediator Drp1 in the organelles of AKI mice.

*In vitro,* in tubular cells SIRT3 overexpression promoted mitochondrial fusion by limiting Drp1 recruitment through the downregulation of Mff and by upregulating Opa1. Moreover, the overexpression of SIRT3 promoted mitochondrial integrity by counteracting the cisplatin-dependent mitochondrial membrane depolarization and translocation of PINK1 on the organelle membrane.

The biological and clinical relevance of SIRT3 in AKI was further documented in SIRT3^−/−^ mice. In these animals, cisplatin induced earlier and remarkably more severe renal dysfunction and, unlike their WT littermates, all SIRT3-deficient mice died within 7 days. SIRT3-deficient animals were fully resistant to the therapeutic effects of both AICAR and ALCAR, which, conversely, were consistently beneficial to SIRT3-competent mice.

Taken together, our results indicate that SIRT3 is a crucial regulator of mitochondrial functional integrity and a non-redundant player in tubular injury and repair in AKI. Given the high prevalence of mitochondrial dysfunction in many chronic diseases, such as metabolic syndrome, premature ageing and cancer, along with the profound impact SIRT3 has on mitochondrial function and metabolism, the obvious translational consequence of these *in vivo* and *in vitro* studies will be to look for potential SIRT3-specific activating compounds, which the current pharmacopeia is remarkably lacking. Recent work has provided evidence that honokiol is capable of blocking cardiac hypertrophy by increasing SIRT3 activity [7], paving the way for disclosing novel SIRT3 activators. Now is the time for academia and industry to jointly tackle this challenge, offering hope to patients who still die of conditions that lack effective treatment.
